# Bioprospecting challenges in unusual environments

**DOI:** 10.1111/1751-7915.12723

**Published:** 2017-06-14

**Authors:** Kristie Tanner, Cristina Vilanova, Manuel Porcar

**Affiliations:** ^1^ Darwin Bioprospecting Excellence SLPaternaSpain; ^2^ I2SysBio (Institute for Integrative Systems Biology)University of Valencia‐CSICPaternaSpain

The microbial ecology field is burgeoning. Year after year, improved sampling, culturing and bioinformatics tools contribute towards an apparently endless increase in microbial diversity. Massive access to genomic data and the development of single‐cell genomic techniques have re‐defined the tree of life by resolving many intra‐ and interphylum level relationships and by including dramatic expansions such as the discovery of a new subdivision in the bacterial domain of life, or an astounding 16‐fold increase in the number of known viral genes (Hug *et al*., 2011; Rinke *et al*., 2013; Paez‐Espino *et al*., 2016). Scaling law‐based calculations have led to the prediction that Earth is the home to more than 1 trillion microbial species (Locey and Lennon, 2016) let alone the intraspecies variation. This is indeed a huge number that may be better internalized with a simple calculation: if scientists were able to summarize in a one‐page genome paper each one of the bacterial species on our planet and piled the 10^12^ resulting pages one on top of another, the total height of the stacked articles would be 100 000 km, approximately a quarter of the distance from the Earth to the Moon. There is no doubt that we are dealing with a terrific amount of microbial diversity, and this puts on the table a double challenge: unveiling the myriad of microbial species still to be discovered and mining such a vast microbial diversity for novel biotechnological tools. Improving current methodologies for the analysis of omic data will be key to detect and identify novel species or gene sequences in massive datasets, whereas new culturing and screening techniques will be needed to exploit their industrial and biomedical applications (Vilanova and Porcar, 2016).

**Figure 1:**
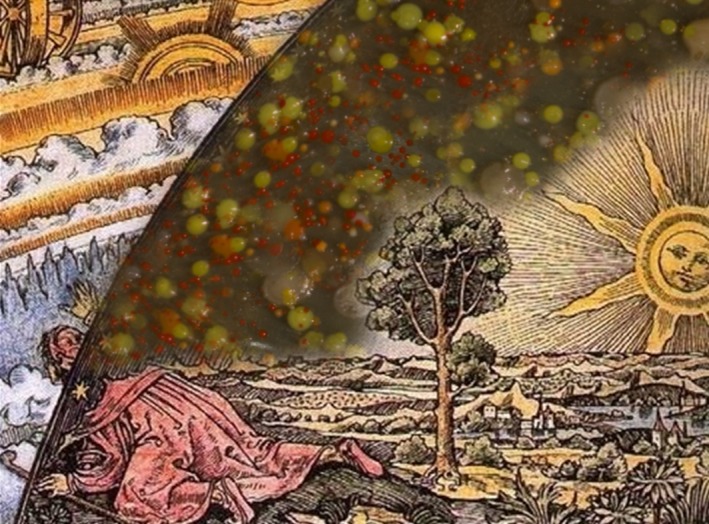
Previous bioprospecting efforts have mainly focused on close environments, but beyond the comfort zone, there exist unexplored unusual niches that hold great promise as a source of biological variation that can have a key role in future biotechnological applications. The image is a collage created by blending bacterial colonies from solar panels (Dorado‐Morales *et al*., 2016) with an adapted version of the famous wood carving ‘Flammarion engraving’ (Flammarion, 1888).

Microbial diversity is everything except random: microorganisms are the result of evolution and adaptation. This provides us with an incredible arsenal of unique and useful pre‐validated tools that can be used in a wide range of industrial applications. The search of these biological tools is what we know as bioprospecting, and it is nothing new. That said, past bioprospecting efforts have mainly focused on close, well‐known environments such as soil, a rich source of antibiotics (Sherpa *et al*., 2015) and bacteria with insecticidal properties (Melo *et al*., 2014); or human gut, from which probiotic bacteria such as *Lactobacillus spp*. can be isolated (Halimi and Mirsalehian, 2016). Nevertheless, exotic, particular environments result in particular adaptations, and the understandable ease with which human or humanized environments can be sampled should not mask that most taxonomic and functional novelties lay somewhere else. Unusual environments remain poorly or unexplored to date although they are certainly valuable sources of novel products. As illustrated by the popular illustration ‘Flammarion engraving’, there is a world, metaphorically, beyond those shining stars we can easily see (Fig. 1).

But, what is an unusual environment, or, more precisely, what is unusual enough? We consider an unusual environment as one that is both poorly explored, taxonomically distant from the human‐associated microbiome and that is under extremophilic conditions. Interestingly, the three features tend to occur at the same time. It has to be stressed that some indoor or outdoor habitats (electrical appliances, sun‐exposed surfaces, high‐temperature saunas) fall in this category.

There are three reasons making unusual environments especially interesting for bioprospecting studies. The first one is the large biodiversity they harbour, leading to a high probability of finding new taxa, as exemplified by the discovery of as many as 47 new phyla in aquifer sediments and groundwater in Colorado (Anantharaman *et al*., 2016). Second, these microorganisms are pre‐adapted to stresses that often correlate with industrial needs. For example, sun‐exposed environments tend to be very rich in pigmented bacteria, such as carotenoid‐producing bacteria on solar panels or scytonemin‐producing bacteria in microbial communities from the Atacama Desert, both of these pigment types with important applications in the food, cosmetic and pharmacological industries thanks to their antioxidant and UV‐protection properties (Vítek *et al*., 2014; Rastogi *et al*., 2015; Dorado‐Morales *et al*., 2016). Finally, a promising research field lies on developing new biofactories from the robust microorganisms able to resist a wide range of stresses (temperature, pH, salinity, etc.). Indeed, bacterial chassis based on *Deinococcus, Hymenobacter, Erythrobacter* and *Geobacillus* species – commonly present in extreme environments like desert soils (Rainey *et al*., 2005), Antarctic environments (Hirsch *et al*., 2004; Kojima *et al*., 2016), spacecraft surfaces (Stepanov *et al*., 2014), the troposphere (DeLeon‐Rodriguez *et al*., 2013), solar salterns (Subhash *et al*., 2013) and mountain peaks (Marchant *et al*., 2002) – are already promising alternatives to classical *E. coli* models for synthetic biology (Gerber *et al*., 2015; Hussein *et al*., 2015).

Biotechnologists are indebted to thermostable polymerases, such as the immensely popular *Taq* polymerase for polymerase chain reactions (PCRs), as well as *Vent* or *Pfu* DNA polymerases, all of them isolated from the extremophilic thermophiles *Thermus aquaticus*,* Thermococcus litoralis* or *Pyrococcus furiosus* respectively (Chien *et al*., 1976; Tindall and Kunkel, 1988; Lundberg *et al*., 1991; Kong *et al*., 1993). There are many other examples of valuable products obtained from unusual environments: from silk from giant riverine orb spiders (Agnarsson *et al*., 2010), to biofuel from hyperthermophilic archaea living in deep‐sea hydrothermal vent chimneys (Nishimura and Sako, 2009), or latex‐degrading bacteria from pine‐tree forests (Vilanova *et al*., 2014). Moreover, the recent development of innovative approaches for the mining of microbial communities is resulting in the discovery of new molecules of outstanding interest. This is the case of *Entotheonella* spp., detected through single‐cell genomics approaches, and producing an unprecedented wide repertoire of bioactive compounds (Wilson *et al*., 2014), or the previously unculturable bacterium *Eleftheria terrae*, isolated from soil with innovative culturing approaches, and producer of the novel antibiotic teixobactin (Ling *et al*., 2015). It is reasonable that improving culturing techniques is first applied to well‐known environments, but they will only be fully exploited on ecologically more ambitious bioprospecting efforts.

Taken together, innovative approaches applied on exotic environments will be the major source of novel microorganisms and/or metabolites in the upcoming future. Taking into account that only a fraction of global microbial diversity has been explored to date (Locey and Lennon, 2016), the number of – yet to be discovered – strains, genetic tools or metabolites with biotechnological or biomedical applications is overwhelming. This opens a great market opportunity for the biotechnology industry and particularly for microbiology‐based enterprises. Highly specialized companies based on the bioprospecting of antibiotics (i.e. Prospective Research, Inc., Beverly, MA, USA) and bioactive molecules from the sea (i.e. Pharmamar, Madrid, Spain), and also new start‐up companies offering improved multi‐omic analysis (i.e. MicrobioMx, Barcelona, Spain) or improved culturing approaches (i.e. Darwin Bioprospecting Excellence) applied to any type of sample, are already part of the bioprospecting marketplace.

During the last two decades, the discovery of novel microbial compounds has declined significantly, mainly as a consequence of the genetic and chemical redundancy detected in commonly analysed environments (Zhang, 2005). Unusual environments hold great promise as unexploited, massively diverse targets for the discovery of biocompounds, microorganisms or consortia with potential commercial and/or industrial applications. We envisage *xenomicrobial bioprospecting* as revolutionary field for both microbial ecologists and entrepreneurs of tomorrow's bioeconomy.
